# The Role of GPR120 Receptor in Essential Fatty Acids Metabolism in Schizophrenia

**DOI:** 10.3390/biomedicines8080243

**Published:** 2020-07-24

**Authors:** Joanna Rog, Anna Błażewicz, Dariusz Juchnowicz, Agnieszka Ludwiczuk, Ewa Stelmach, Małgorzata Kozioł, Michal Karakula, Przemysław Niziński, Hanna Karakula-Juchnowicz

**Affiliations:** 1 1st Department of Psychiatry, Psychotherapy and Early Intervention, Medical University of Lublin, 20-439 Lublin, Poland; hanna.karakula-juchnowicz@umlub.pl; 2 Chair of Chemistry, Department of Analytical Chemistry, Medical University of Lublin, 20-093 Lublin, Poland; anna.blazewicz@umlub.pl (A.B.); pnizinski11@gmail.com (P.N.); 3 Department of Psychiatric Nursing, Medical University of Lublin, 20-093 Lublin, Poland; juchnowiczdariusz@wp.pl; 4 Independent Laboratory of Natural Products Chemistry, Department of Pharmacognosy, Medical University of Lublin, 20-093 Lublin, Poland; agnieszka.ludwiczuk@umlub.pl; 5 2nd Department of Psychiatry and Psychiatric Rehabilitation, Medical University of Lublin, 20-439 Lublin, Poland; ewastelmach@umlub.pl; 6 Chair and Department of Medical Microbiology, Medical University of Lublin; 20-093 Lublin, Poland; malgorzata.koziol@umlub.pl; 7 Student Research Team from Department of Analytical Chemistry, Medical University of Lublin, 20-093 Lublin, Poland; michal.karakula@gmail.com; 8 Department of Clinical Neuropsychiatry, Medical University of Lublin, 20-439 Lublin, Poland

**Keywords:** G protein-coupled receptors, GPR120, FFAR4, schizophrenia, polyunsaturated fatty acids, long-chain fatty acids, omega-3, nutritional psychiatry

## Abstract

A growing body of evidence confirms abnormal fatty acid (FAs) metabolism in the pathophysiology of schizophrenia. Omega-3 polyunsaturated fatty acids (PUFAs) are endogenous ligands of the G protein-coupled receptors, which have anti-inflammatory properties and are a therapeutic target in many diseases. No clinical studies are concerned with the role of the GPR120 signaling pathway in schizophrenia. The aim of the study was to determine the differences in PUFA nutritional status and metabolism between patients with schizophrenia (SZ group) and healthy individuals (HC group). The study included 80 participants (40 in the SZ group, 40 in the HC group). There were no differences in serum GPR120 and PUFA concentrations and PUFA intake between the examined groups. In the HC group, there was a relationship between FAs in serum and GPR120 concentration (*p* < 0.05): α-linolenic acid (ALA) (*R* = −0.46), docosahexaenoic acid (DHA) (*R* = −0.54), omega-3 PUFAs (*R* = −0.41), arachidonic acid (AA) (*R* = −0.44). In the SZ group, FA serum concentration was not related to GPR120 (*p* > 0.05). In the HC group, ALA and DHA serum concentrations were independently associated with GPR120 (*p* < 0.05) in the model adjusted for eicosapentaenoic acid (EPA) and accounted for 38.59% of GPR120 variability (*p* < 0.05). Our results indicate different metabolisms of FAs in schizophrenia. It is possible that the diminished anti-inflammatory response could be a component connecting GPR120 insensitivity with schizophrenia.

## 1. Introduction

Polyunsaturated fatty acid (PUFA) imbalance is linked with various clinical conditions, especially neuropsychiatric diseases, including schizophrenia [1]. Numerous reports confirmed that patients with schizophrenia (SZ) have lower levels of blood omega-3 fatty acids (FAs) compared with healthy individuals [2,3]. The comparison of the erythrocyte FA composition in 429 subjects with schizophrenia and 444 healthy individuals revealed that the patients had lower levels of omega-3 PUFAs fatty acids: docosahexaenoic acid (DHA, 22:6), docosapentaenoic acid (DPA, 22:5), eicosapentaenoic acid (EPA, 20:5), and an omega-6 fatty acid, arachidonic acid (AA, 20:4) [4]. The mechanism of undernutrition of PUFAs in the SZ population is still under examination. Studies indicated that several factors engage in improper FA blood concentrations, including poor nutrition, antipsychotic drug interactions with cell membranes, and disturbances of lipid metabolism, as well as fatty acid-dependent signaling pathways [2,3,5].

The clinical findings support the abnormal metabolism of FAs in SZ, reflected by attenuation of responses to the niacin skin flush test. The dermal application of aqueous methyl nicotinate (AMN) leads to redness, as well as edema of skin as a result of the cascade of inflammatory reactions mediated by phospholipase A2 (PLA2) [6,7]. PLA2 activates the release of omega-6 AA from membrane phospholipids which is related to various physiological processes connected with emotions, motivation, response to stress, and energy homeostasis, including eicosanoid biosynthetic enzymes, as well as promotion of the immune type 2 response and initiation of endocannabinoid signaling in the brain [8,9].

PLA2 activity can be modulated by a G-coupled receptor responsive to fatty acids—GPR120 (also known as an free fatty acid receptor 4: FFAR4). Omega-3 PUFAs are endogenous ligands of the receptor. FA stimulation leads to GPR120–β-arrestin-2 complex formation and contributes to anti-inflammatory signaling pathway activation [10]. GPR120 is a therapeutic target in many diseases, especially diabetes and other inflammatory conditions [11]. Taking into account lipid disturbances in schizophrenia and the mechanistic and genetic connection between schizophrenia and inflammatory disorders, GPR120 could also be a promising target in this condition [12]. An animal study showed that receptor activation in the microglia of the hypothalamus reduces neuroinflammation via a decrease in pro-inflammatory cytokine synthesis [13]. However, little is known about GPR120 in psychiatric disorders. To our best knowledge, no clinical studies are concerned about the role GPR120 signaling together with FA metabolism in schizophrenia.

**The aim** of the study was to determine the differences in PUFAs nutritional status and metabolism between patients with schizophrenia and healthy individuals. More specifically, we examined and compared serum concentrations of GPR120, polyunsaturated fatty acids (omega-6: AA, omega-3: DHA, EPA, α-linolenic acid (ALA)) and dietary intake of omega-3 and omega-6 FAs (omega-6: LA—linolenic acid (precursor of AA)), omega-3: DHA, EPA, ALA) between patients and healthy individuals. The secondary aim was to find a relationship between PUFA metabolism and clinical/sociodemographic variables in the examined population.

## 2. Materials and Methods

### 2.1. Study Participants

The age of eligible participants ranged between 18 and 65. Forty outpatients suffering from schizophrenia (SZ group) according to the Diagnostic and Statistical Manual of Mental Disorders (DSM-5) criteria [14] were recruited in the study. Out of them, 95% were treated with antipsychotic medication. Forty healthy volunteers matched for age and body mass index (BMI) were enrolled in the study, as the control (HC group). Participants from the HC group had no psychiatric, inflammatory-related, metabolic disorders, or other health problems which in the examiner’s opinion could have affected FA metabolism, while they were also not taking any medication. Neither group followed any specific diet within the six months prior to the examination. Before entering the study, all subjects gave their informed consent to participate. The study was conducted in accordance with the Declaration of Helsinki [15], and the protocol was approved by the Ethics Committee of Medical University of Lublin, Poland (Project identification code: KE-0254/127/2016, 28 April 2016).

### 2.2. Blood Collection

Venous blood (10 mL) samples were collected after overnight fasting using S-Monovette^®^ 4.9 mL and a Clotting Activator/Serum blood collection system (Sarstedt, Nümbrecht, Germany). Serum was obtained by centrifugation (at 2000× *g*, 10 min) and stored at −80 °C for later analyses (but no longer than a six-month period). No hemolysis was observed in any of the samples.

### 2.3. Fatty Acids Assay

Serum samples were thawed and a mixture of chloroform/methanol (2:1 *v*/*v*) was added to a 50-μL aliquot. Samples were flushed with nitrogen gas and stored at 4 °C. The next step was to perform double extraction. After drying, lipids (in lower phase) were saponified (using a mixture of KOH and methanol) and subsequently methylated by boron trifluoride (14%). The analysis was carried out using gas chromatography–mass spectrometry (GC/MS) (Shimadzu GC-2010 PLUS gas chromatograph coupled to a Shimadzu QP2010 Ultra mass spectrometer (Shim-pol, Warsaw, Poland)). Compounds were separated on a fused-silica capillary column SP^TM^-2560 (100 m, 0.25 mm inner diameter (i.d.)) with a film thickness of 0.20 mm (Supelco, Darmstadt, Germany). The injection volume was 1.0 μL, and the temperature of the injection port was 230 °C with a split ratio of 1:20, while helium was used as a carrier gas; the temperature program was 70 °C/2 min, 175 °C/25 min, and 200 °C/17 min. As done by other authors, non-endogenous C17:0 free fatty acid (5 μg of 1 mg/mL stock) was used as an internal standard [16].

### 2.4. GPR120 Assay

GPR120 serum concentration was assessed using a commercially available kit (O3FAR1 ELISA Kit, EIAab, Gentaur Poland, Sopot, Poland) according to the manufacturer’s instruction. The sensitivity of the test was 0.097 ng/mL. The number of studies focusing on membrane proteins in serum is still limited. Nevertheless, this type of analysis has the potential to diagnose and/or treat diseases in further clinical practice [17].

### 2.5. Dietary Assessment

The intake of FAs was assessed during a face-to-face interview by a registered dietitian (J.R.) using the 24 h recall method referring to the day prior blood collection. The nutritional value of the diet was determined using nutrition analysis software (ESHA Food Processor SQL, version 10.1.1; ESHA, Salem, OR, USA) with additional Polish Food composition tables (the standard reference food composition database of nutrients in foods and dishes commonly consumed in Poland) [18].

### 2.6. Sociodemographic and Clinical Data

All participants answered questions via a structural interview. The self-created questionnaire was always filled out by the same person. The clinical data of patients were obtained from a supervising physician. The questionnaire was composed of the following parts: sociodemographic/anthropometric information, lifestyle (including dietary habits), medical data (in case of patients, including duration of illness, number of hospitalizations, using medication).

### 2.7. The Severity of Schizophrenia Symptoms Assessment

The severity of schizophrenia symptoms was assessed using the Polish adaptation of the Positive and Negative Symptom Scale (PANSS) by a well-trained physician [E.S] [19]. The examination was always performed on the same day assessing other variables.

### 2.8. Statistical Analysis

Statistical analyses were conducted using Statistica software (TIBCO Software Inc., Palo Alto, CA, USA). The Shapiro–Wilk test was performed to explore variable distribution. To determine differences between groups, a chi-square test for categorical variables and a Mann–Whitney U-test for continuous variables were used. To determine the magnitude and direction of the correlation between examined variables, Spearman’s rho correlation was used. The multiple-step regression analysis was carried out to explain the variability in GPR120 concentration depending on the nutritional status or sociodemographic data. For all analyses, a value of *p <* 0.05 was considered statistically significant. When multiple statistical tests were performed, Bonferroni correction was applied [20].

## 3. Results

### 3.1. Study Participant Characteristics

The sociodemographic and clinical characteristics of the examined group are presented in Table 1. The study included 40 individuals with schizophrenia (SZ group; mean age: 31 years old; 52.5% males) and 40 healthy volunteers as the control (HC group) (mean age: 29 years old; 37.5% males). There were no significant differences between the clinical and control groups in age, gender, and body mass index (BMI) (*p* > 0.05). The median of illness duration was 78 months, and the number of hospitalizations was two. In total, 95% of patients were taking antipsychotic medication. Most of the SZ group was treated with second-generation antipsychotic drugs (*n* = 36; 90%). Other patients (*n* = 2; 5%) received first-generation antipsychotic drugs, one of them together with an anticonvulsant drug. In total, five persons (12.5%) received first-generation treatment, 13 (32.5%) individuals took anticonvulsant medications, and six individuals (15%) received selective serotonin reuptake inhibitors (SSRIs). Two patients (5%) received benzodiazepines. Furthermore, 5% (*n* = 2) of the SZ group was antipsychotic-free due to medication nonadherence. The median modal doses of antipsychotic medication treatment were 15 mg of olanzapine equivalent [21]. The average severity of schizophrenia symptoms measured with the Positive and Negative Symptom Scale (PANSS) scale was 54 points (median) (a maximum score of 210 points).

### 3.2. Nutritional Status and Metabolism of PUFAs

Table 2 shows the FA and GPR120 concentrations in SZ and HC groups. There were no significant differences in the concentrations of AA, ALA, EPA, DHA, total omega-3, PUFAs, and GPR120 between patients and the control group.

As it was shown in Table 2, the examined groups had a similar intake of total fat, as well as omega-3 and omega-6 PUFAs. The mean intake of EPA + DHA was 88.17 mg and 34.86 mg in the SZ and HC groups, respectively, and the same number of participants from both groups (97.5%) had an intake of omega-3 FAs below the recommended daily intake (RDA; 250 mg) [22].

### 3.3. Effect of Nutritional Status on PUFA Metabolism

**Figure 1:**
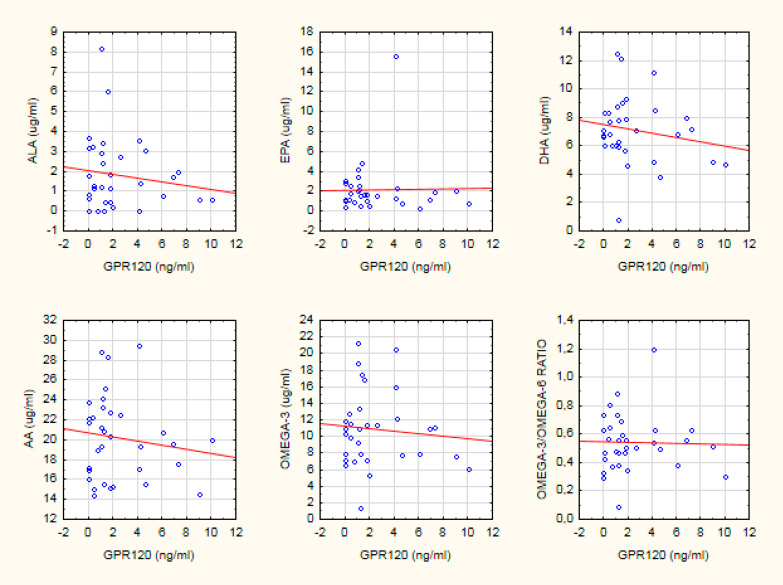
The scatter plot of the relationship between GPR120 and PUFA serum concentration in the SZ group. Spearman’s rank correlation coefficient was calculated.

In the HC group, there was a relationship between FAs in serum and GPR120 concentration. ALA (*R* = −0.46; *p* < 0.05), AA (*R* = −0.44; *p* < 0.05), DHA (*R* = −0.54, *p* < 0.05), and omega-3 FA (*R* = −0.41; *p* < 0.05) concentrations were inversely associated with GPR120. In the SZ group, FA serum concentration was not related to GPR120 (*p* > 0.05) (see Table 3, and Figure 1). The revealed relationships were not significant after Bonferroni correction for multiple comparisons (*p* > 0.0083).

In the patient group, there were only positive relationships between EPA intake and serum FA concentration: ALA (*R* = 0.46; *p* < 0.05), AA, (*R* = 0.44; *p* < 0.05), DHA (*R* = 0.46; *p* < 0.05), and omega-3/omega-6 PUFA ratio (*R* = 0.35; *p* < 0.05) (see Table 4).

**Figure 2:**
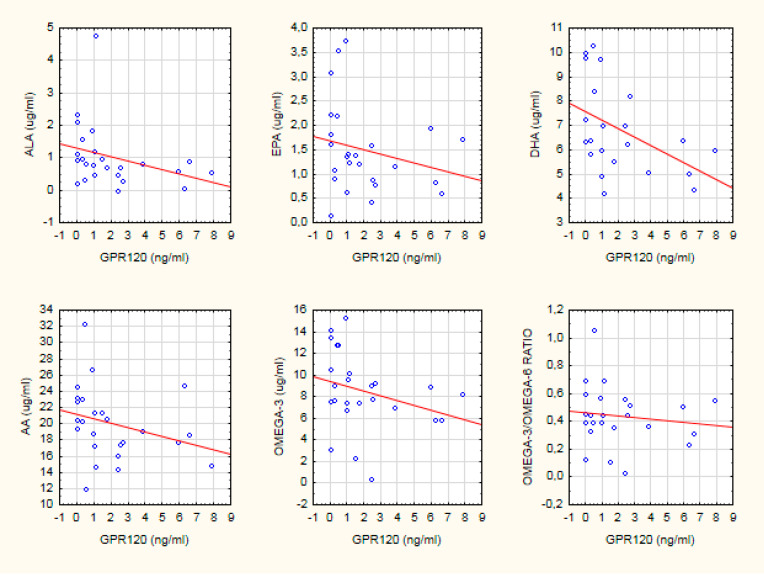
The scatter plot of the relationship between GPR120 and PUFA serum concentration in the HC group. Spearman’s rank correlation coefficient was calculated.

There were various correlations between FA intake and their concentration in serum in the HC group (see Figure 2). The serum concentration of DHA was positively associated with the intake of PUFAs (total amount) (*R* = 0.40; *p* < 0.05), LA (*R* = 0.40; *p* < 0.05), and omega-6 PUFAs (*R* = 0.39; *p* < 0.05) (see Table 4). A higher intake of DHA was associated with a lower serum concentration of ALA (*R* = −0.34; *p* < 0.05).

However, the relationships were not significant after Bonferroni correction for multiple comparisons (*p* > 0.0017).

### 3.4. Effect of Demographic and Clinical Variables on PUFAs Nutritional Status

In the SZ group, there was a positive correlation between DHA intake and BMI (*R* = 0.39; *p* < 0.05), as well as between duration of illness and BMI (*R* = 0.38; *p* < 0.05). We did not find a relationship between schizophrenia symptoms (measured with PANSS) or clinical data and FA nutritional status or metabolism.

In the HC group, there was a positive relationship between AA serum concentration and age (*R* = 0.36; *p* < 0.05) and BMI (*R* = 0.44; *p* < 0.05), between EPA concentration and BMI (*R* = 0.33; *p* < 0.05), and between DHA concentration and age (*R* = 0.41; *p* < 0.05). There was also an inverse relationship between GPR120 serum concentration and age (*R* = −0.50; *p* < 0.05) and between intake of EPA and age (R = −0.45; *p* < 0.05), as well as a positive relationship between intake of LA and BMI (*R* = 0.44; *p* < 0.05) and between intake of total PUFAs and BMI (*R* = 0.40; *p* < 0.05).

### 3.5. GPR120 Serum Concentration Variability

To further determine the independent predictors of GPR120 serum concentration using a multiple regression model, the following explanatory variables were used: age, BMI, and serum concentration of ALA, AA, EPA, and DHA. In the patient group, there were no relationships between GPR120 concentration and other variables (*p* > 0.05). In the HC group, ALA and DHA serum concentration were independently associated with GPR120 (*p* < 0.05) in a model adjusted for EPA concentration. The model explained 38.59% of GPR120 serum concentration variability. The estimated relationship was not statistically significant after Bonferroni correction (*p* > 0.0167).

## 4. Discussion

An increasing amount of evidence confirms the role of abnormal lipid metabolism in the pathobiology and clinical course of schizophrenia [2,3,4]. Despite the fact that research concerning fatty acid metabolism is going on for a long time, there is an insufficient amount of evidence to determine the mechanism and all pathways engaged in lipid disturbances related to schizophrenia [23]. Differences in PUFA levels between patients suffering from schizophrenia and healthy individuals were also reported [4]. Until now there are no guidelines for the routine assessment of serum FA levels or concentrations in patients with psychiatric illness.

**Figure 3:**
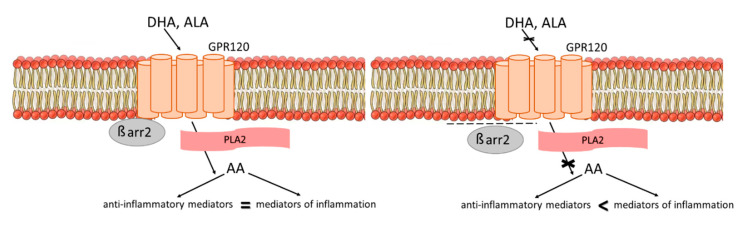
The proposed mechanism of the connection between GPR120 insensitivity and impaired lipid metabolism in schizophrenia. In healthy individuals, GPR120 activation leads to anti-inflammatory effects. Natural ligands (DHA and ALA) stimulate GPR120–β-arrestin complex formation and drive phospholipase A2 (PLA2) activation. Pro-/anti-inflammatory homeostasis is maintained. In schizophrenia patients, GPR120 insensitivity leads to pro/anti-inflammatory imbalance. Natural ligands (DHA and ALA) are unable to stimulate GPR120–β-arrestin complex formation. Overactivity of PLA2 causes a switch to inflammatory pathway stimulation. Pro-/anti-inflammatory homeostasis is disturbed.

In our study, there were no differences in GPR120 levels between patients and healthy individuals. Nevertheless, the negative relationship between GPR120 serum concentration and DHA and ALA concentration was detected only among healthy individuals. We did not find any correlation between GPR120 receptor concentration and FA serum concentration in the SZ group. This phenomenon suggests that patients suffering from schizophrenia may manifest a GPR120 insensitivity with activation becoming impossible (see Figure 3). The revealed relationships were found to be insignificant after Bonferroni correction for multiple comparisons. To some extent, the small sample size could have also affected the lack of relationship after performing multiple comparisons. Further studies with a larger sample size are required.

It is still unclear whether GPR120 concentration in the serum reflects the expression of the receptor and cell signaling. We decided to examine the serum concentration of GPR120 for some reasons. Firstly, this type of sample is more stable during storage in low temperatures compared to RNA [24]. Gene testing is essential to confirm the results of our study. Introducing serum sample collection in clinical practice from a population health perspective is more convenient and accessible compared to RNA samples. Thus, serum samples may be a more favorable material used as a biomarker of fatty acid metabolism disturbances in further clinical practice [17,25].

In a population of children, lower GPR120 plasma levels were associated with negative outcomes, i.e., insulin resistance and higher BMI [26]. In our study, we did not find any relationship between BMI and GPR120, and lower GPR120 serum concentration was related to the higher concentration of PUFAs in the HC group. It is possible that a higher concentration of PUFAs in serum increases anti-inflammatory status, and GPR120 activation (to restore balance) is not required. On the other hand, an activated GPR120 forms a complex with β-arrestin, which may lead to a reduction in free GPR120 blood concentration [10].

The mechanism linked with GPR120 disruption in schizophrenia may be related to PLA2 activity. According to Horrobin’s theory of schizophrenia, PLA2 excess activity is linked with an abnormal skin reaction in the niacin test in schizophrenia patients [27]. PUFAs are able to activate PLA2 via the GPR120 receptor, leading to the production of prostaglandin. This pathway is involved in the anti-inflammatory DHA effects in macrophages [28].

GPR120 activation mediates downstream signaling mechanisms and prevents the expression of proinflammatory cytokines. Thus, GPR120 has the potential to diminish systemic inflammation and manage metabolic functions [10,12]. There is a suggestion that several beneficial effects of omega-3 do not require GPR120. However, GPR120 function is essential to regulate vascular inflammation and neointimal hyperplasia [29]. Disruption of the anti-inflammatory GPR120-related pathway could affect the persistent proinflammatory state in schizophrenia and may be involved in a higher risk of cardiometabolic conditions in the psychiatric population [30]. However, GPR120 participation in dyslipidemia observed in schizophrenia patients remains unclear.

Clinical trials showed the ability of omega-3 PUFAs to reduce clinical symptoms of schizophrenia [31,32]. We did not find any differences in PUFA concentration and intake between schizophrenia patients and healthy individuals. The pharmacological treatment of schizophrenia increases the sterol regulatory element-binding protein type 1 (SREBP1) which regulates the expression of genes related to fatty acid synthesis [33]. This phenomenon to a certain degree may explain the lack of difference in serum FAs between SZ and HC groups. Antipsychotic medication increases the expression and activity of enzymes involved in PUFA metabolism, and changes in the blood plasma lipidome are suggested as a treatment response in psychiatric disorders [5]. On the other hand, in our study, the nutritional assessment concerned short-term omega-3 intake. This short period of dietary habit analysis was performed due to the evaluation of serum as a biomarker of PUFAs. We hypothesize that the severity of symptoms will be more linked to long-term omega-3 intake. However, in patients suffering from schizophrenia, especially those who experienced psychotic symptoms recently, examining long-term intake will be a challenge. People with schizophrenia commonly experience cognitive impairment, lack of motivation, and poor compliance; thus, misreporting could be expected, especially regarding long-term food intake assessment [34,35].

Adequate intake of omega-3 fatty acids plays an important role in maintaining mental health. DHA and EPA are structurally integrated via phospholipid molecules and they ensure the proper structure of neuronal cell membranes [3]. FAs regulate the expression of genes, as well as changes in protein concentration, which could be blocked to some extent in SZ patients [36].

Each omega-3 PUFA has various molecular effects, and EPA levels are several hundred-fold lower than DHA levels [37]. Studies confirmed the anti-inflammatory potential of DHA. However, DHA formulas did not improve schizophrenia symptoms, which suggest an abnormal or lack of response to its supplementation [38]. GPR120 insensitivity and changes in FAs metabolism could, to some extent, explain the better efficacy of EPA in SZ. In our study, we found numerous correlations between the intake of EPA and the concentration of blood PUFAs in patients, which we did not notice in the HC group. These findings may be the result of a different utilization of FAs in metabolic pathways.

Increasing ALA while simultaneously decreasing LA intake is effective in improving omega-3 PUFA status [39]. The scientific community suggests that the dietary ratio of omega-6/omega-3 PUFAs should be from 2:1 to 4:1; however, in the Western diet, the ratio ranges from 15:1 to 17:1 [39]. In our study, 85% of patients and 80% of healthy individuals had a ratio higher than 4:1, and the median ratio was 6.23:1 in the SZ group and 5.23:1 in the HC group. Taking into consideration the variation PUFA metabolism between patients and healthy subjects, it is possible that individuals with psychiatric disorders require a higher intake or different proportions of omega-3/omega-6 FAs for health benefits. The different recommended intake of PUFAs in the psychiatric population should be considered [40]. No differences in the intake of PUFAs between persons with schizophrenia and healthy volunteers was revealed. Nevertheless, we found improper intake of DHA and EPA in 97.5% of the SZ group and 97.5% of the HC group.

## 5. Conclusions

Our consistent findings report lipid disturbances in schizophrenia patients. Interestingly, no changes in serum FAs or related markers were found. However, our results indicate different transformations and responses to FAs in schizophrenia. Based on our results, it is suggested that a diminished anti-inflammatory response could be a component connecting GPR120 insensitivity with schizophrenia. The interplay involving the inflammatory processes confirmed in psychiatric diseases, the limited ability to extinguish them, and the imbalance in PUFA diet could lead to the worsening course of schizophrenia, with increasing metabolic complication risk.

Further work should concentrate on finding lipid-based biomarkers and lipid-related interventions in patients suffering from schizophrenia. The modulation of lipid homeostasis is a promising target in managing psychiatric disorders.

## 6. Advantages and Limitations

The study has some potential advantages and limitations. To the best of our knowledge, this is the first work examining GPR120 protein concentration in schizophrenia and the relationship between GPR120 with PUFAs. Fatty acids were determined using a very sensitive and modern analytical technique (GC/MS) that enables accurate and precise measurements even in a very complex sample matrix such as human serum.

The study has some limitations. The sample size was relatively small and we mainly examined patients treated with antipsychotic drugs and in remission. Further studies should concentrate on lipid metabolism at a different stage of illness and include antipsychotic-naïve patients.

Another disadvantage is the dietary assessment using a single 24 h recall. Although it is considered the least biased self-report tool, three or more daily recalls are needed to determine the usual intake [41]. Through contact loss with most of the respondents, we were unable to apply multiple daily recalls.

The obtained data could be considered only as a possible explanation for the lipid disruption in SZ. An examination of gene expression involved in fatty acid metabolism, along with their variants and the main metabolites of PUFAs, is needed to confirm this hypothesis. The quality and the quantity of FAs in the erythrocyte membrane are considered more appropriate markers of the long-term nutritional status of the entire organism. Nevertheless, red blood cell FAs are more inclined to undergo deterioration during storage [42].

We included potential confounding factors related to lifestyle (BMI, age). However, taking into consideration the complexity and interactions of metabolic pathways, the examination of processes affecting the presence of substrates in blood (related to gene expression, enzyme activity) is necessary to determine the exact mechanism and importance of lipid metabolism in schizophrenia [43].

## Figures and Tables

**Table 1:** The characteristics of studied population.

Clinical Data	Schizophrenia (*n* = 40)		Healthy Controls (*n* = 40)		SZ vs. HC
	Mean (Median)	SD	Mean (Median)	SD	
Age	31 (30)	7.32	29 (27)	7.93	NS
BMI (kg/m^2^)	26.6 (26.6)	5.12	24.6 (24.2)	4.39	NS
Duration of illness (months)	90 (78)	83.43	NA	NA	NA
Number of hospitalization	2.7 (2)	2.25	NA	NA	NA
Olanzapine equivalents	18.12 (15)	13.93	NA	NA	NA
PANSS total	55.35 (54)	26.71	NA	NA	NA

PANSS—Positive and Negative Symptom Scale; SZ—schizophrenia; HC—healthy control; BMI—body mass index; SD—standard deviation; NA—not applicable; NS—not significant. The Mann–Whitney U-test was used.

**Table 2:** The nutritional fatty acid status and metabolism.

	Schizophrenia (*n* = 40)		Healthy Controls (*n* = 40)		SZ vs. HC
	Mean (Median)	SD	Mean (Median)	SD	
**Serum Measurement**					
ALA (mcg/mL)	1.61 (1.16)	1.74	0.91 (0.67)	0.93	NS
EPA (mcg/mL)	2.03 (1.37)	2.45	1.45 (1.38)	0.89	NS
DHA (mcg/mL)	6.88 (6.81)	2.26	6.80 (6.41)	1.91	NS
AA (mcg/mL)	19.63 (19.48)	4.10	19.92 (19.36)	3.75	NS
PUFAs (mcg/mL)	29.80 (28.61)	7.62	28.57 (27.19)	5.76	NS
Omega-3 (mcg/mL)	10.18 (10.05)	4.50	8.65 (8.34)	3.27	NS
Omega-3/6 ratio ^1^	0.52 (0.50)	0.20	0.44 (0.45)	0.18	NS
GPR120 (ng/mL)	2.41 (1.24)	2.72	2.00 (1.02)	2.32	NS
**Dietary Assessment**					
Fat (g)	81.11 (78.63)	34.65	85.62 (80.26)	39.04	NS
PUFAs (g)	14.01 (10.69)	8.20	12.33 (10.84)	6.22	NS
Omega-3 (g)	2.33 (1.42)	2.78	1.87 (1.66)	1.04	NS
Omega-6 (g)	11.67 (9.35)	7.25	10.45 (9.40)	5.56	NS
18:2 LA (g)	11.55 (9.23)	7.24	10.34 (9.35)	5.51	NS
20:5 EPA (mg)	35.45 (0)	187.63	6.21 (0)	17.54	NS
22:6 DHA (mg)	52.72 (10)	177.18	28.65 (10)	71.07	NS

^1^ Expressed as a (DHA + EPA + ALA)/AA concentration; PUFAs—polyunsaturated fatty acids; ALA—α-linolenic acid; EPA—eicosapentaenoic acid; DHA—docosahexaenoic acid; AA—arachidonic acid; GPR120—G protein-coupled receptor 120; LA—linolenic acid; SZ—schizophrenia; HC—healthy control; SD—standard deviation; NS—not significant. The Mann–Whitney U-test was used.

**Table 3:** The relationship between GPR120 and PUFA serum concentration.

GPR120	ALA	EPA	DHA	AA	Omega-3	Omega-3/6 Ratio ^1^
Healthy controls	−0.46 *	NS	−0.54 *	−0.44 *	−0.41 *	NS
Schizophrenia	NS	NS	NS	NS	NS	NS

^1^ Expressed as a (DHA + EPA + ALA)/AA concentration; * *p* < 0.05; NS—not significant. Spearman’s rank correlation coefficient was calculated.

**Table 4:** Relationship between PUFA intake and their metabolism.

	Blood	ALA	EPA	DHA	AA	Omega-3/6 Ratio ^1^	GPR120
Diet							
**SZ group**							
20:5 EPA		0.46 *	NS	0.46 *	0.44 *	0.35 *	NS
**HC group**							
PUFAs		NS	NS	0.40 *	NS	NS	NS
Omega-6		NS	NS	0.39 *	NS	NS	NS
18:2 LA		NS	NS	0.40 *	NS	NS	NS
22:6 DHA		−0.34 *	NS	NS	NS	NS	NS

^1^ Expressed as a (DHA + EPA + ALA)/AA concentration; * *p* < 0.05; NS—not significant. Spearman’s rank correlation coefficient was calculated.

## Abbreviations

PUFAs	Polyunsaturated fatty acids
FAs	Fatty acids
DHA	Docosahexaenoic acid
DPA	Docosapentaenoic acid
EPA	Eicosapentaenoic acid
AA	Arachidonic acid
SZ	Schizophrenia
AMN	Aqueous methyl nicotinate
PLA2	Phospholipase A2
GPR120	G-coupled receptor responsive to fatty acids
LA	Linolenic acid
ALA	α-Linolenic acid
HC	Healthy control
BMI	Body mass index
PANSS	Positive and Negative Symptom Scale
RDA	Recommended daily intake
SREBP1	Sterol regulatory element-binding protein type 1
DSM-5	The Diagnostic and Statistical Manual of Mental Disorders
GS/MS	Gas chromatography–mass spectrometry
